# Volumetric Optoacoustic Temperature Mapping in Photothermal Therapy

**DOI:** 10.1038/s41598-017-09069-5

**Published:** 2017-08-29

**Authors:** Francisco Javier Oyaga Landa, Xosé Luís Deán-Ben, Ronald Sroka, Daniel Razansky

**Affiliations:** 1 Institute for Biological and Medical Imaging (IBMI), Helmholtz Center Munich, Neuherberg, Germany; 2 Faculty of Medicine, Technical University of Munich, Munich, Germany; 3 Laser Research Laboratory/LIFE Center, Ludwig-Maximilian-University, Munich, Germany

## Abstract

Photothermal therapy and ablation are commonplace medical procedures employed for treatment of tumors, vascular and brain abnormalities as well as other disorders that require selective destruction of tissues. Yet, accurate mapping of the dynamic temperature field distribution in the treated region represents an unmet clinical need, strongly affecting the clinical outcome of these interventions. We introduce a fast three-dimensional temperature mapping method based on real-time optoacoustic sensing of the treated region coupled with a thermal-diffusion-based model of heat distribution in tissues. Deviations of the optoacoustic temperature readings provided at 40  ms intervals remained below 10% in tissue-mimicking phantom experiments for temperature elevations above 3 °C, as validated by simultaneous thermocouple measurements. Performance of the new method to dynamically estimate the volumetric temperature distribution was further showcased in *post-mortem* mouse imaging experiments. The newly discovered capacity to non-invasively measure the temperature map in an entire treated volume with both high spatial and temporal resolutions holds potential for improving safety and efficacy of light-based therapeutic interventions.

## Introduction

Thermal therapies are widely employed in clinical practice, from selective ablation of cancerous tissues, benign hyperplasias and varicose veins to elimination of subcutaneous fat, cardiac arrhythmias and enhanced drug delivery^1–6^. Several sources have been considered for the heating purposes, among them laser light, focused ultrasound, radio-frequency current and microwaves^2^. Laser-induced thermotherapy (LITT), also referred to as laser ablation, has gained popularity due to its important advantages, such as minimal invasiveness, low hardware costs and reduced treatment time^3, 4^. LITT employs laser radiation as an energy source, often guided through optical fibers to the target tissue as e.g. in percutaneous laser ablation (PLA) or endovenous laser therapy (ELT). In addition, light-absorbing agents are often used to target photothermal procedures and further serve as theranostic agents^5–7^. For example, semiconducting polymer nanobioconjugates have been recently suggested as theranostic amplifiers for combined optoacoustic (OA) imaging and photothermal therapy^8^ as well as for targeted photothermal activation of neurons^9^.

Both the temporal and the spatial temperature distribution in the treated tissue play a crucial role in the outcome of photothermal interventions. Heat-driven denaturation is generally facilitated when tissues are heated above 50 °C, while the exposure time further determines the size of the induced lesion. Yet, several therapeutic procedures use lower temperature elevations without inducing irreversible tissue damage, including local and whole-body hyperthermia^10^ as well as low- and medium-intensity focused ultrasound^11, 12^. The effectiveness of thermal therapies thus heavily relies on the ability to closely monitor and control the volumetric temperature distribution of the treated tissues in real time^2^. Invasive approaches based on thermocouples or fiber-optic sensors^13^ can be used for temperature monitoring. In this way, the temperature can only be captured in a few locations within the heated region while a direct tissue contact or an invasive approach are further required. On the other hand, the temperature map can in principle be obtained using non-invasive imaging modalities such as optical methods^14, 15^, infrared thermometry^16^, ultrasound^17^, x-ray computed tomography (CT)^18^, or magnetic resonance imaging (MRI)^19^. However, these techniques are either limited by low penetration, sensitivity, contrast or otherwise lack an adequate temporal resolution for dynamic mapping of the temperature fields.

OA imaging may potentially represent an advantageous approach for monitoring phototherapy due to its high sensitivity to temperature variations^20–24^. The temperature dependence of the OA signal during thermal therapies has been previously established^25–28^. Yet, no three-dimensional (3D) mapping of the temperature field in real time has been so far demonstrated. Herein, we study the application of optoacoustics to map the temperature distribution during laser-induced thermal therapy. In particular, evolution of volumetric OA data is correlated with thermocouple readings in controlled tissue-mimicking phantom experiments in order to establish the lower boundaries on the accuracy of the temperature estimations. A thermal-diffusion-based model of heat distribution in tissues is further employed in order to render the spatio-temporal distribution of the temperature field in experiments performed in highly heterogeneous mouse tissues.

## Methods

### Optoacoustic data acquisition set-up and image reconstruction

**Figure 1:**
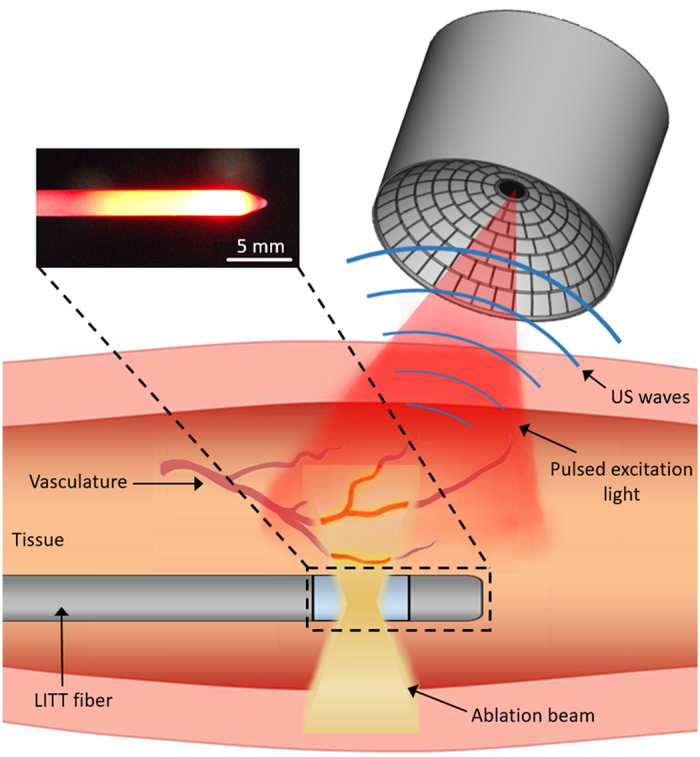
Lay-out of the experimental setup. The drawings of the transducer array and the ablation system were developed by the authors.

The lay-out of the experimental setup is shown in Fig. 1. Briefly, heating was induced using a diode laser generating 20 W of continuous wave power at a wavelength of 830 nm (Indigo 830, Indigo Medical Inc., Lawrenceville, New Jersey). A specialized fiberoptic delivery system with a cylindrical diffuser at the tip was used to guide the ablation beam to the region of interest. Volumetric temperature monitoring was performed with a 3D OA imaging system consisting of a 512-element spherical transducer array covering an angle 140° with 4  cm radius of curvature (1.3π solid angle). The individual elements of the array have a central frequency of 5  MHz and ~100% detection bandwidth, corresponding to nearly isotropic imaging resolution of ~150  µm around the geometrical center of the sphere. Acoustic coupling was ensured by molding agarose gel between the active surface and the surface of the imaged sample (Fig. 1). OA responses were excited with a short-pulsed (~8 ns) laser source (Innolas Laser GmbH, Krailling, Germany) guided via a custom-made fiber bundle (CeramOptec GmbH, Bonn, Germany) through a hollow cylindrical cavity in the center of the array. For imaging, the wavelength of the tunable optoacoustic laser source was also set to 830 nm and the optical fluence was approximately 11  mJ/cm^2^ at the surface of the imaged sample. The pulse repetition frequency (PRF) of the laser was set to 5  Hz. A second arm of the fiber bundle was guided to a Coherent powermeter EM-USB-J-25MB-LE (Coherent Inc., Santa Clara, California) to monitor the energy per pulse, which was used to normalize the acquired signals. All 512 OA signals were simultaneously digitized at 40 mega-samples per second (MSPS) by a custom-made data acquisition (DAQ) system (Falkenstein Mikrosysteme GmbH, Taufkirchen, Germany) triggered with the Q-switch output of the laser.

OA images were subsequently reconstructed with a graphics processing unit (GPU)-based 3D back-projection algorithm^29^. Prior to reconstruction, the acquired signals were deconvolved with the impulse response of the array elements and band-pass filtered between 0.1 MHz and 7 MHz.

### Temperature Estimation Method

Our OA temperature estimation is based on the temperature dependence of the thermoelastic conversion efficiency. When the OA signals are excited with a short-duration laser pulse, the so-called stress-confinement conditions can be assumed for the initial OA signal (pressure) distribution *p*_0_ in the medium^30^. In this case, the latter is given via *p*_0_ = *Γμ*_*a*_ *Φ*, being Γ the (dimensionless) Grüneisen parameter, *µ*_a_ the optical absorption coefficient and *Φ* the light fluence. The temperature dependence of the generated OA signals mainly comes from variations in the Grüneisen parameter, whose dependence on temperature in water-like aqueous solutions can be approximated by^30^

1 $${\Gamma }(T)=0.0043+0.0053T,$$

where *T* is expressed in °C. Eq. (1) describes temperature dependence of the Grüneisen parameter for water and diluted aqueous solutions, which was verified with empirical measurements across a wide range of temperatures^31^. The relative change of the OA signal as a function of the temperature increase ΔT can be then expressed as

2 $$\frac{{\rm{\Delta }}{p}_{0}}{{p}_{0,0}}=\frac{0.0053\,{\rm{\Delta }}T}{0.0043+0.0053{T}_{0}},$$

being *p*_0,0_ and *T*_0_ the initial (baseline) optoacoustic signal and the initial temperature before the ablation experiment, respectively. According to Eq. 2, the amplitude of the OA signals is expected to increase by approximately 2.7% per degree for typical temperature values of 36 °C in living organisms. We further define *F* as the ratio between the relative increment of the OA signal and the relative increment of temperature, i.e.,

3 $$F=\frac{{T}_{0}}{{p}_{0,0}{\rm{\Delta }}T}{\rm{\Delta }}{p}_{0}$$

Considering Eq. 2, the theoretical value of *F* (*F*_*th*_) can be expressed as a function of the initial temperature *T*_0_ via

4 $${F}_{th}={(\frac{0.8113}{{T}_{0}}+1)}^{-1}$$

The temperature increment can then be estimated from the relative OA signal increase as

5 $${\rm{\Delta }}T=\frac{{T}_{0}}{{p}_{0,0}{F}_{th}}{\rm{\Delta }}{p}_{0}$$

It should be noted that, unlike for inorganic liquids, protein denaturation and coagulation processes are known to take place in soft biological tissues for temperatures exceeding 50 °C, introducing significant additional complexity due to non-linear alterations of the Grüneisen parameter^26^ as well as alterations in the optical absorption and scattering coefficient of the ablated tissues^32^. Accuracy of the above temperature estimation approach is therefore expected to be limited to the temperatures range lying below the coagulation threshold.

### Phantom validation experiments

The quantitative performance of the temperature monitoring method according to Eq. 5 was experimentally tested in a tissue-mimicking phantom. The phantom consisted of three tubings with 1 mm diameter and 10 mm length embedded in a ~8 mm thick layer of chicken breast. The tubings were filled with murine blood and sealed with glue. Three thermocouples (Physitemp Instruments Inc., Clifton, New Jersey) were inserted into the tubings to provide real-time temperature values. The thermocouple readings were digitized with an embedded NI 9213 DAQ (National Instruments Corporation, Austin, Texas, U.S.). For each tubing, the temperature was estimated from the OA signal in an ROI corresponding to the exactly known location of each thermocouple.

### The Thermal Diffusion Model

High resolution temperature mapping in real highly heterogeneous tissues represents a much more challenging task than pointwise temperature estimations. This is chiefly because the temperature elevation due to laser-induced heating depends on both the local light fluence distribution and the optical absorption coefficient. As a result, a highly non-uniform temperature distribution is expected to occur in heterogeneous tissues with many locations exhibiting low absorption or otherwise insignificant temperature alterations that cannot be accurately estimated by optoacoustics. We therefore assumed that only voxels that generated OA signals above a certain threshold, set to 20% of the maximum signal in the entire 3D image volume, can be effectively used for reliable temperature estimations, whereas the remaining voxels are assumed to be mainly affected by heat diffusion from the adjacent voxels exhibiting higher absorption values. A model based on the heat diffusion equation has been thus introduced to dynamically estimate the volumetric temperature distribution via^33^

6 $$\frac{\partial T(r,t)}{\partial t}-\,D{\nabla }^{2}T(r,t)=\,\frac{Q(r,t)}{\rho \,{C}_{p}},$$

where *T*(*r*,*t*) is the spatio-temporal map of the temperature increase, *Q*(*r*,*t*) is the heat absorbed in the tissue per unit volume and unit time, *ρ* is the tissue mass density, *C*_*p*_ is its specific heat capacity per unit mass, and *D*=*k*/*ρC*_p_ is the thermal diffusivity, being *k* the thermal conductivity in [W/m °C]. We assumed *ρ = 1.06 g/ml, C*_*p*_ *=3.5 J/gK, D = 1.14∙10* ^*–7*^ *m* ^2^ */s* for soft biological tissues^33^. The Green’s solution to Eq. (6) is given by

7 $$T(r,t)={\int }_{0}^{t}{\int }_{V}\frac{Q(r,t)}{\rho {C}_{p}}\,g(r,t,r{\text{'}},t{\text{'}})\,dr{\text{'}}dt{\text{'}},$$

where g(*r, t, r′, t′*) is given by^33^

8 $$g(r,t,r{\text{'}},t{\text{'}})=\,\frac{1}{{(4\pi D(t-t{\text{'}}))}^{3/2}}\,\cdot exp(-\frac{{|r-r{\text{'}}|}^{2}}{4D(t-t{\text{'}})}).$$

Eq. (7) is subsequently approximated by assuming that energy absorption takes place at a finite number of points and time instants, leading to

9 $$T(r,t)\approx \sum _{i,j}{T}_{i,j}(r,\,t),\,$$

where

10 $${T}_{ij}(r,\,t)=\,\frac{{E}_{i}({t}_{j})}{\rho {C}_{p}}\,(\frac{1}{{(4\pi D(t-{t}_{j}))}^{3/2}})\cdot exp(\frac{-{|r-{r}_{i}|}^{2}}{4D(t-{t}_{j})}).$$

The spatial locations *r*_i_ and temporal instants *t*_j_ correspond to the position of the reconstructed voxels and the optoacoustic sampling instants, respectively. *E*_i_(*t*_j_) represents the amount of energy absorbed in the *i*-th voxel for the *j*-th time interval (between *t*_j_-*Δt* and *t*_j_), i.e.,

11 $${E}_{i}({t}_{j})=\,\rho V{C}_{p}{\rm{\Delta }}{T}_{i}({t}_{j}),$$

where *V* is the volume corresponding to each reconstructed voxel and Δ*T*_i_(*t*_j_) is the temperature rise at the *i*-th voxel during the *j*-th interval. Eq. (10) is then rewritten as

12 $${T}_{ij}(r,\,t)=V{\rm{\Delta }}{T}_{i}({t}_{j})\,(\frac{1}{{(4\pi D(t-{t}_{j}))}^{3/2}})\cdot exp(\frac{-{|r-{r}_{j}|}^{2}}{4D(t-{t}_{j})}).$$

On the other hand, for voxels whose values exceeded 20% of the maximum value in the entire 3D image volume the temperature was solely estimated by relying on the instantaneous OA signal values *T*(*r*_i_, *t*_j_) without considering thermal diffusion, i.e.,

13 $${\rm{\Delta }}{T}_{i}({t}_{j})=T({r}_{i},{t}_{j})-T({r}_{i},{t}_{j}-{\rm{\Delta }}t).$$

### Mouse experiments

The performance of the suggested temperature mapping approach was tested experimentally in a mouse that was ablated *post mortem*. Specifically, the ablation fiber was rectally inserted with the mouse lying in a supine position, so that the OA laser illuminated the back of the mouse. The initial (ambient) temperature of the mouse was *T*_0_ =22 °C. The ablation laser was stopped after 36 s and the optoacoustic images were acquired from the heated area over 120 s, thus also covering the cooling phase. All experiments were performed in full compliance with the institutional guidelines of the Helmholtz Center Munich and with approval from the Government District of Upper Bavaria. Note that a large number of thermocouples would be required for validating the temperature mapping results in 3D in the highly heterogeneous murine tissue while accurate positioning of the thermocouples in the intact mouse is technically challenging. Thus, no thermocouple validation was performed in this case and the experiment was done in order to showcase temperature monitoring in a real heterogenous biological specimen.

## Results

### Tissue-mimicking phantom experiments

**Figure 2:**
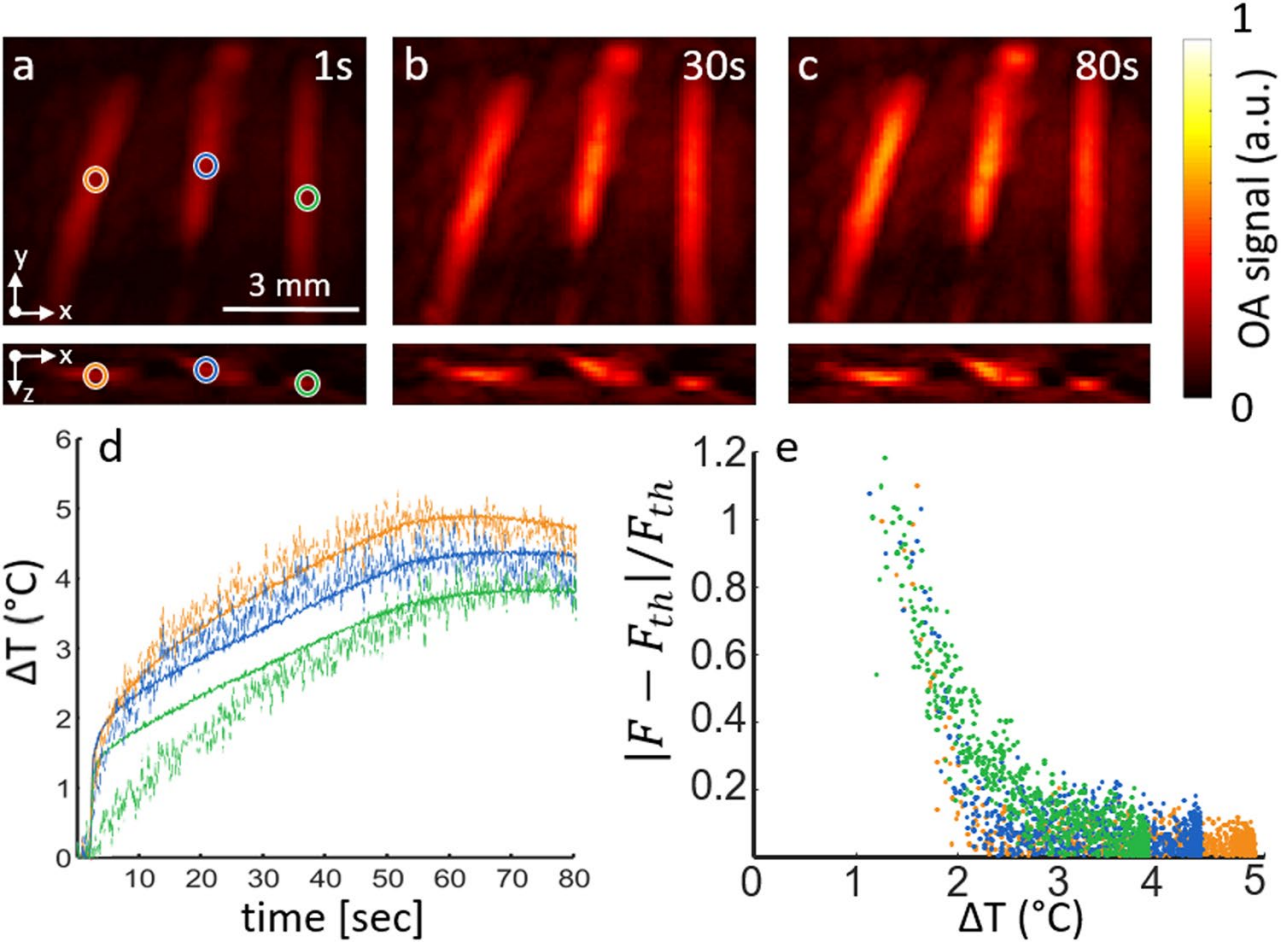
Optoacoustic temperature estimations in a tissue mimicking phantom. (**a**–**c**) Transverse and coronal maximum intensity projection (MIP) optoacoustic images reconstructed for three different time points during laser heating of the phantom; (**d**) The temperature increase estimated from the optoacoustic signal variations (dashed curves) as compared to the temperature increase measured with thermocouples (solid curves). The regions of interest considered for the estimation are marked in panel a; (**e**) Relative deviation of the *F* factor from the theoretical value as a function of the temperature increase.

Figure 2 shows the OA images captured at three different instants during the laser heating procedure. The ablation fiber was aligned parallel to the tubings at a distance of approximately 5 mm from the leftmost one. A progressive increase in the OA signal intensity as the temperature rises is clearly visible in all tubings (Fig. 2a–c). The temperature increase in blood was then estimated from Eq. 5, where the *F* factor is calculated according to Eq. 4. The estimated temperature increase inside the three tubings is plotted in Fig. 2d (dashed lines). For calculating the relative signal increments, a reference OA image (the baseline image) was taken as the average of 50 frames immediately preceding the ablation procedure. The temperature increase values measured with the thermocouples (located approximately at the same ROIs) are also shown in Fig. 2d (continuous lines).

As expected, lower temperature increments result in larger disagreement between the optoacoustically-estimated temperature values and those measured with the thermocouples. Figure 2e indicates that the relative deviations in *F* remain below 10% if the temperature increases by at least 3 °C. These deviation can be partially attributed to the relative uncertainty in the measured Δ*p*_0_ values (see Eq. 3), originating from the noise in OA measurements. Note however that the discrepancy may also result from inaccuracies in the theoretical *F* values (*F*_th_) that were calculated using Eq. 4 assuming a homogenous water medium. Additional uncertainty exists in the location of the thermocouple tip, which may not exactly coincide with the locations where the representative OA traces were analyzed, as labeled by circles in Fig. 2a.

### Mouse experiments

**Figure 3:**
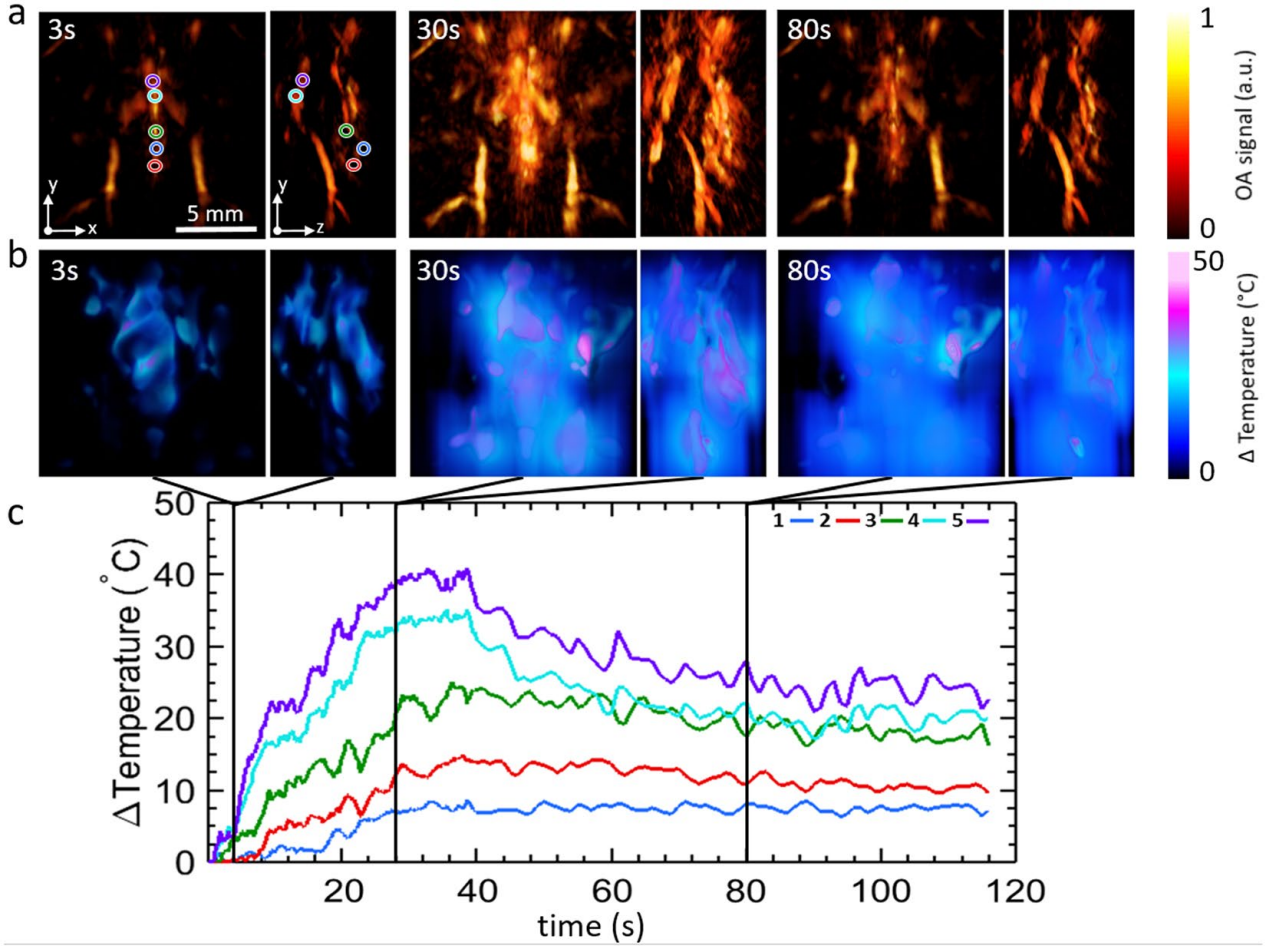
Volumetric optoacoustic monitoring of temperature during photothermal therapy performed in a mouse *post mortem*. (**a**) Transverse and lateral MIPs of the reconstructed optoacoustic volumes at different time points during the treatment. The tip of the ablation fiber is located approximately at the center of the displayed volumes. (**b**) Transverse and lateral MIPs of the estimated temperature maps at the same time points. (**c**) Actual temporal traces of temperature from the points labeled in (**a**).

Figure 3a shows the reconstructed OA images (MIPs along the transverse and sagittal views) for three different instants during photothermal therapy performed in a mouse *post mortem*. Clearly, the OA images deliver 3D maps of the mouse anatomy at high spatial resolution in the 150 µm range while also exhibiting rapid OA signal variations closely following the temperature rise. The dynamic temperature maps estimated via the methodology described in the Methods section are shown in Fig. 3b. Plots in Fig. 3c depict the temporal evolution of the temperature profiles at 5 different locations, as indicated in Fig. 3a. The gradual heating and thermal diffusion effects can be best perceived in a video showing real-time monitoring of the temperature distribution in the mouse (Supplementary Video 1). It can be observed that points closer to the ablation fiber experience more pronounced temperature rise in the first few seconds following initiation of the ablation procedure and reach the maximum temperature approximately when the ablation laser is switched off. On the other hand, lower peak temperatures and slower temperature changes are exhibited at locations farther away from the fiber (e.g. points 1, 2 and 3). Yet, the temperature in those regions remains nearly constant after the heating is terminated, indicating that thermal diffusion plays a dominant role in maintaining the energy balance in those regions.

## Discussion

The lack of simple and reliable non-invasive temperature feed-back represents a major barrier towards broader adaptation of laser-based thermotherapy procedures in pre-clinical research and clinical routine. The presented results showcase that volumetric OA imaging may emerge as a promising tool for quantitative monitoring of the temperature field during thermal therapies. Optoacoustics is particularly suitable for this purpose due to its high sensitivity to the temperature changes as well as the powerful ability to represent the temperature changes in an entire treated volume with both high spatial and temporal resolutions in the 150 µm and 10 ms ranges, respectively.

Note that in the experiments performed, the temperature dependence of the Grüneisen parameter was adopted from an empirical formula for diluted aqueous solutions, which may not accurately represent the physical reality in soft biological tissues^26^. Thus, accurate calibration of this parameter in different tissues may result in better accuracy when estimating the temperature-dependence of the OA signals. In addition, accuracy of the temperature estimations has been shown to be directly linked to the contrast and noise levels of the reconstructed optoacoustic images while the average optoacoustic signal strength is expected to drop by approximately an order of magnitude for each centimeter of penetration in living tissues at the near-infrared wavelengths^30^. In the current study, temperature estimations were achieved at up to 10 mm depth in *ex-vivo* mouse tissues without employing signal averaging. For monitoring at deeper locations, one potential solution may involve guiding the OA excitation beam through the same fiber used for delivering the ablation beam. In this way, since both OA imaging and laser ablation are usually performed in the near-infrared wavelength range, an even higher level of hardware integration could be potentially achieved if the same laser is used for both ablation and generation of OA responses.

The amount of monitored information can be enhanced by acquisition of multispectral optoacoustic tomography (MSOT) data^34^. Since MSOT enables identifying spectral variations in the imaged tissue, this information may further help improving the accuracy of the suggested temperature monitoring approach by recognizing alterations in the optical or chemical parameters of the imaged tissue resulting from e.g. tissue coagulation.

In the present study, the underlying modeling assumption was that the tissue optical properties remain unchanged during the heating process, which is not the case if tissue coagulation or other irreversible thermal damage occur. It has been previously observed that a stronger and non-linear dependence of the OA signal intensity on temperature was produced in tissues heated above 53 °C^26^. In addition, not only the Grüneisen parameter but several other physical parameters, such as optical absorption and scattering, are altered due to tissue coagulation^34^. As a result, solutions of the OA inversion and thermal diffusion problems in three and four dimensions become much more complicated in this case and require a different mathematical treatment, which will be addressed in future work. During *in-*vivo thermal therapy treatments, it is further expected that the results are affected by alterations in blood perfusion induced by tissue heating. Indeed, a higher blood volume may lead to an additional OA signal increase not related to the temperature changes. On the other hand, increased blood perfusion also has a cooling effect^2, 35^, which may result in further inaccuracies introduced into the suggested thermal diffusion model.

## Conclusion

In conclusion, we introduced a new high resolution volumetric temperature monitoring method based on real-time acquisition of three-dimensional optoacoustic data coupled with thermal-diffusion-based model of heat distribution in tissues. The results suggest that the proposed approach is capable of mapping the development of the temperature field during laser-induced thermal therapies, which can potentially contribute to improving safety and efficacy of these treatments.

## Electronic supplementary material

Supplementary Video Information

Volumetric Optoacoustic Temperature Mapping in Photothermal Therapy
