# Supplementary material for: Volumetric Optoacoustic Temperature Mapping in Photothermal Therapy
Source: Sci Rep. 2017 Aug 29;7:9695. doi: 10.1038/s41598-017-09069-5 (PMC5575057; doi:10.1038/s41598-017-09069-5)
Supplement: Supplementary file 1 — Supplementary Video Information [file 41598_2017_9069_MOESM1_ESM.doc]

A supplementary video file is included with the following article

Title: Volumetric Optoacoustic Temperature Mapping in Photothermal Therapy

Authors: Francisco Javier Oyaga Landa, Xosé Luís Deán-Ben, Ronald Sroka and Daniel Razansky

**Supplementary Video Caption.** Real-time optoacoustic temperature monitoring during photothermal therapy. Left: Volumetric optoacustic image sequence. Right: Volumetric sequence of the estimated temperature maps during laser ablation.
